# Statistical evaluation of test-retest studies in PET brain imaging

**DOI:** 10.1186/s13550-018-0366-8

**Published:** 2018-02-12

**Authors:** Richard Baumgartner, Aniket Joshi, Dai Feng, Francesca Zanderigo, R. Todd Ogden

**Affiliations:** 1 Merck and Co., Inc., Kenilworth, NJ USA; 2 Novartis Institutes for Biomedical Research, Cambridge, USA; 3 Department of Psychiatry, Columbia University Medical Center, New York, NY USA; 4 Molecular Imaging and Neuropathology Division, New York State Psychiatric Institute, New York, NY USA; 5 Department of Biostatistics, Mailman School of Public Health, Columbia University, New York, NY USA

## Abstract

**Background:**

Positron emission tomography (PET) is a molecular imaging technology that enables in vivo quantification of metabolic activity or receptor density, among other applications. Examples of applications of PET imaging in neuroscience include studies of neuroreceptor/neurotransmitter levels in neuropsychiatric diseases (e.g., measuring receptor expression in schizophrenia) and of misfolded protein levels in neurodegenerative diseases (e.g., beta amyloid and tau deposits in Alzheimer’s disease). Assessment of a PET tracer’s test-retest properties is an important component of tracer validation, and it is usually carried out using data from a small number of subjects.

**Results:**

Here, we investigate advantages and limitations of test-retest metrics that are commonly used for PET brain imaging, including percent test-retest difference and intraclass correlation coefficient (ICC). In addition, we show how random effects analysis of variance, which forms the basis for ICC, can be used to derive additional test-retest metrics, which are generally not reported in the PET brain imaging test-retest literature, such as within-subject coefficient of variation and repeatability coefficient. We reevaluate data from five published clinical PET imaging test-retest studies to illustrate the relative merits and utility of the various test-retest metrics. We provide recommendations on evaluation of test-retest in brain PET imaging and show how the random effects ANOVA based metrics can be used to supplement the commonly used metrics such as percent test-retest.

**Conclusions:**

Random effects ANOVA is a useful model for PET brain imaging test-retest studies. The metrics that ensue from this model are recommended to be reported along with the percent test-retest metric as they capture various sources of variability in the PET test-retest experiments in a succinct way.

## Background

Positron emission tomography (PET) is a molecular imaging technology used for in vivo measurement of metabolism and neurochemistry, including measurement of cerebral blood flow, glucose metabolism, oxygen utilization, and density of neuroreceptors or other molecular targets [1, 2]. As an integral component of the validation of novel PET tracers, a test-retest experiment is usually first conducted to measure repeatability of the measurements.

The main purpose of a test-retest experiment is to inform about within-subject variability, i.e., how close the measurements are when they are obtained repeatedly on the same subject under identical conditions. It is common then to compare these measures of repeatability—certainly, when considering multiple methods of processing and/or modeling PET data. Often, standardized measures of repeatability are used as general metrics to help judge the general utility of a tracer, although it is not obvious that it is appropriate to compare these measures across tracers or across molecular targets.

The test-retest experiment is most naturally relevant for evaluating a tracers’ utility for use in a study involving multiple measurements on the same subject, e.g., an occupancy study or a study measuring the effect of some intervention. As we will summarize here, most of the indices used to summarize the results of test-retest experiments measure quantities that are important for such experiments. Note, however, that these indices by themselves do not provide all the useful information when considering other types of PET studies, i.e., a cross-sectional study of two groups of subjects.

Still, the test-retest repeatability of a tracer is an important criterion to help select a tracer for a particular target among multiple available tracers [3], although of course several other criteria (e.g., robust radiochemistry, large specific-to-nonspecific signal, and absence of off-target binding) are also important factors. Going beyond tracer evaluation, test-retest studies also provide useful data for determining the optimal approach among various quantification techniques (e.g., modeling strategies or outcome measures) for a given tracer. Test-retest studies are also useful for understanding the relative variability among multiple region of interests (ROIs).

In general, test-retest repeatability usually refers to measuring the variability when repeated measurements are acquired on the same experimental unit under identical (or nearly identical) conditions [4]. Various metrics have been proposed in the statistical and PET literature to evaluate test-retest experiments such as percent test retest (PTRT), intraclass correlation coefficient (ICC), within-subject coefficient of variation (WSCV) or repeatability coefficient (RC), and we will describe these in some detail in the next section. Briefly, these metrics can be classified as either scaled or unscaled indices of agreement [5]. Unscaled indices of agreement summarize the test-retest repeatability based on differences of original measurements and therefore are obtained on the original unit of measurement, example of which would be RC. In contrast, scaled indices of agreement are normalized with respect to some given quantity and are therefore (unitless) relative measures. Common examples of scaled measures are “percent test retest” which is commonly reported in PET studies.

A very recent article by Lodge [6]*,* assesses repeatability of very common PET-based measurements in oncology applications focusing on only one tracer (^18^F–FDG) and one summary measure (standardized uptake value (SUV)). In that paper, Lodge reviews multiple relevant test-retest studies that report results in inconsistent ways depending on several repeatability measures, and so syntheses of these studies is quite challenging. This illustrates the need to critically evaluate the various measures that are reported in the PET imaging literature. Our objective here is to provide a comprehensive assessment of test-retest evaluations in PET brain imaging, in particular with respect to the assumptions of the random effects ANOVA model that underlies the ICC statistic. Similar critical reviews of repeatability experiments have recently been conducted for other modalities (e.g., electrocardiogram data [7]). To illustrate the utility of the different test-retest metrics, we reevaluated data from five published brain PET test-retest studies in humans. Finally, we provide a discussion of the merits and applicability of the test-retest metrics for future PET brain imaging studies.

## Methods

### Description of the data sets

We considered five published brain PET test-retest datasets [8–12], whose details are reported in Table 1.

**Table 1:** Summary table of the considered clinical brain PET test-retest data sets

Data set	Data set ID	Study	Target	Number of subjects
[^11^C]CUMI-101	DS1	Milak et al., J Nucl Med. 2010; 51(12): 1892–900	Serotonin 1A receptor	7
[^11^C]DASB	DS2	Ogden et al., J Cereb Blood Flow Metab. 2007; 27(1): 205–17	Serotonin transporter	10
[^11^C]PE2I	DS3	Delorenzo et al., J Cereb Blood Flow Metab. 2009; 29(7): 1332–45	Dopamine transporter	7
[^11^C]WAY-100635	DS4	Parsey et al., J Cereb Blood Flow Metab. 2000; 20(7): 1111–33	Serotonin 1A receptor	5
[^11^C]ABP688	DS5	Delorenzo et al., J Cereb Blood Flow Metab. 2011; 31(11): 2169–80	Glutamate receptor subtype 5	8

### Statistical model for test-retest

The most basic model for a test-retest experiment is the standard random effects ANOVA:

1 $$ {y}_{ij}=\mu +{s}_i+{e}_{ij} $$

where *y*_*ij*_ is the PET outcome measure corresponding to scan *j* observed on the *i-*th subject (*i* = 1…*n*) (typically two repeated scans (*j* = 1,2) are obtained in brain PET test-retest studies), *s*_*i*_ is the subject-level random effect, and *e*_*ij*_ is the measurement error, with *s*_*i*_ and *e*_*ij*_ mutually independent and normally distributed:

$$ {\displaystyle \begin{array}{l}{s}_i\sim N\left(0,{\sigma}_s^2\right)\kern1em \\ {}{e}_{ij}\sim N\left(0,{\sigma}_e^2\right)\end{array}} $$

where *σ*_*s*_and *σ*_*e*_are the between- and within-subject standard deviations, respectively.

Estimation of the parameters μ, σ_e_, and σ_s_ in model (1) is described in Appendix for completeness. The computation was implemented using the R package “agRee” [13]*.* There are two scaled indices and one unscaled index of agreement that naturally ensue from model (1) that were proposed for characterization of a test-retest experiment:

1) the WSCV [14, 15], defined as

2 $$ \mathrm{WSCV}=\frac{\sigma_e}{\mu } $$

2) the ICC, defined as

3 $$ \mathrm{ICC}=\frac{\sigma_S^2}{\sigma_S^2+{\sigma}_e^2} $$

3) an unscaled RC, that is given as

4 $$ \mathrm{RC}=\sqrt{2}\kern0.5em {z}_{1-\alpha /2}\kern0.5em {\sigma}_e, $$

where *z*_1−α/2_ is the 1−α/2 quantile of standard normal distribution. The RC can also be interpreted as the smallest detectable difference (SDD) between a test and retest measurement for a given subject. It is defined as a 100(1−α/2)% quantile of the distribution of test-retest differences. Thus, this quantile represents limits of a typical range containing large proportion (e.g., 95%) of the distribution of test-retest differences (with α = 0.05, *z*_1−α/2_ = 1.96 [15]).

As described in the “Introduction” section, percent test-retest (PTRT) is a ubiquitous measure in PET brain imaging although it is not often used in other related fields. In early PET test-retest papers, signed (or raw) mean normalized test-retest differences were considered [16, 17], but later authors generally used the absolute values of the normalized differences instead [18]. Following this latter definition, PTRT is calculated as follows:

5 $$ \mathrm{PTRT}=\frac{1}{n}\sum \limits_{i=1}^n\left|2\frac{y_{i2}-{y}_{i1}}{y_{i2}+{y}_{i1}}\right| $$

Where *n* is the number of subjects in the test-retest study and *y*_*i1*_ and *y*_*i2*_ are the estimated PET outcome measures obtained for the *i-*th subject in a given region in the test and in the retest scan, respectively.

### Bland-Altman plot

Bland-Altman plots show mean vs. difference of test-retest observations for each subject involved in the study and therefore provide a comprehensive visual assessment of the data [19]*.*

### PET test-retest data

The total volume of distribution (*V*_*T*_) [20] was considered as the PET outcome measure that was calculated using three different quantification strategies, one- (1TC) and two-tissue compartment (2TC) models [21], and a graphical approach, the likelihood estimation in graphical analysis (LEGA) [22].It should be noted that the purpose here of considering three different quantification approaches is not to revisit the question of determining the “best” modeling approach for each tracer. This question has been adequately addressed in the original manuscripts for the respective tracers. Rather, multiple quantification approaches provide additional datasets to illustrate how the different test-retest metrics can be applied and what attributes of the data and quantification method can be measured. Ten ROIs were considered in common across all five data sets: anterior cingulate, amygdala, dorsal caudate, dorsolateral prefrontal cortex, gray matter cerebellum, hippocampus, insula, midbrain, parietal lobe, and ventral striatum. In the case of [^11^C]WAY-100635, an additional ROI, the white matter cerebellum, was considered [11], but not included in this analysis to maintain the same ROIs across all tracers*.* The test-retest variability is a result of noise in the ROI and in the arterial input function and is impacted by the size of the ROI. Analysis in this paper does not consider the ROI size as a factor, since ROI-size is the same for different tracers binding to the same target.

## Results

**Fig. 1:**
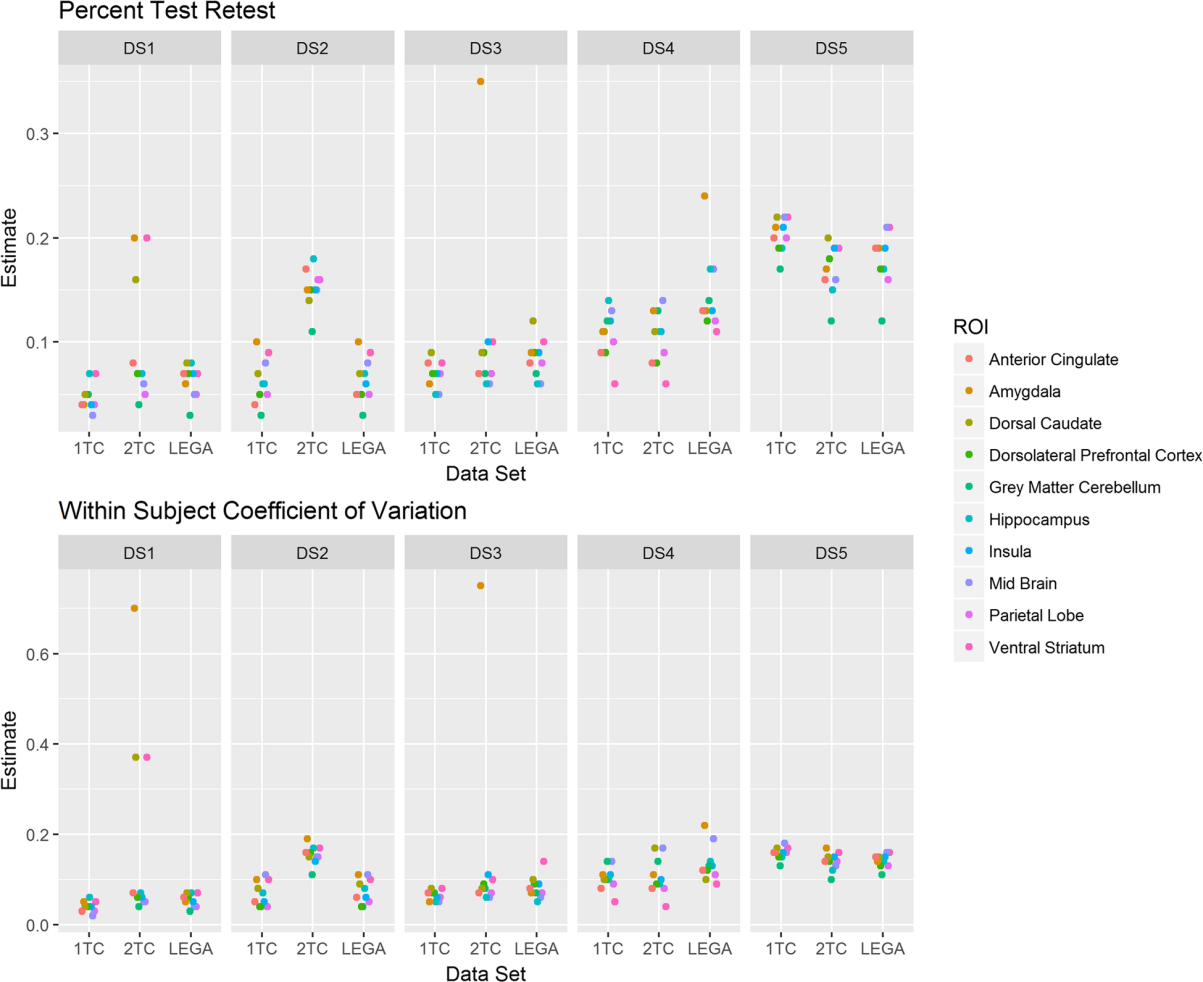
Percent test-retest (PTRT, upper panel) and within-subject coefficient of variation (WSCV, lower panel) across the considered datasets. Each point in the plot, for a given dataset, represents a particular region of interest (ROI) so that the plot represents the variability of PTRT or WSCV across the considered ROIs. For each data set, DS1, DS2, DS3, DS4, and DS5, there were three quantification methods investigated (1, 2, and L denote one-tissue compartment model, two-tissue compartment model, and likelihood estimation in graphical analysis, respectively)

The variability of PTRT and WSCV across datasets and considered ROIs is shown in Fig. 1. For a given dataset, each point in this plot represents a particular ROI. Both metrics show similar values (between 5 and 20%) for most datasets and for the majority of ROIs. Whether the test-retest metric of any given tracer is adequate for any particular clinical study depends on the effect size being investigated. Among the relevant ROIs, the ROIs with better test-retest reliability will be typically used for the main analysis. For some datasets, measures of reliability may be different depending on the selected modeling approach. As an example, results for the DS1 dataset ([^11^C]CUMI-101) are summarized below. According to the PTRT and WSCV criteria, for most datasets, the 2TC model shows worse test-retest reliability than the more parsimonious 1TC model, as expected. Graphical approaches (such LEGA) tend to be more robust than kinetic models to presence of noise in the data, and thus usually yield fewer or no outliers, which can influence test-retest repeatability. Among kinetic models, the 2TC model is more prone to generating outliers than the more parsimonious 1TC model. To demonstrate how various ROIs are performing across different test-retest metrics, they are plotted in the same color across datasets and fitting methods.

**Fig. 2:**
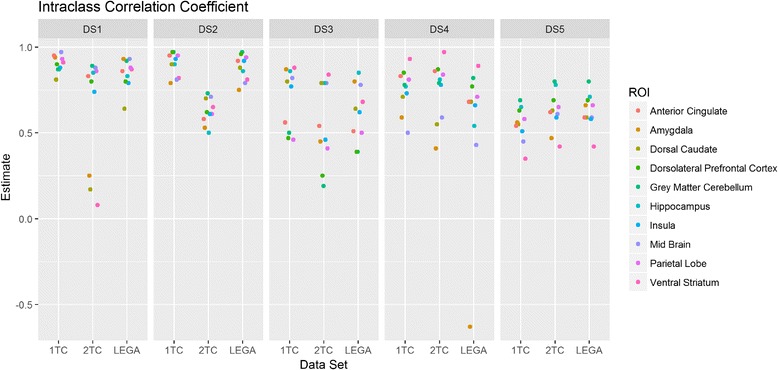
Intraclass correlation coefficient (ICC) across the considered datasets. Each point in the plot, for a given dataset, represents a particular region of interest (ROI) so that the plot represents the variability of ICC across the considered ROIs. For each data set, DS1, DS2, DS3, DS4, and DS5, there were three quantification methods investigated (1, 2, and L denote one-tissue compartment model, two-tissue compartment model, and likelihood estimation in graphical analysis, respectively)

The ICC values obtained across datasets (shown in Fig. 2 in the same fashion as in Fig. 1) provide a similar picture as PTRT and WSCV in terms of test-retest repeatability. The ICC ranges between very high (close to 1) and lower (ICC value ~ 0.5). Again, outlying ROIs for the 2TC model in datasets DS1 and DS3 considerably reduce the corresponding ICC.

**Fig. 3:**
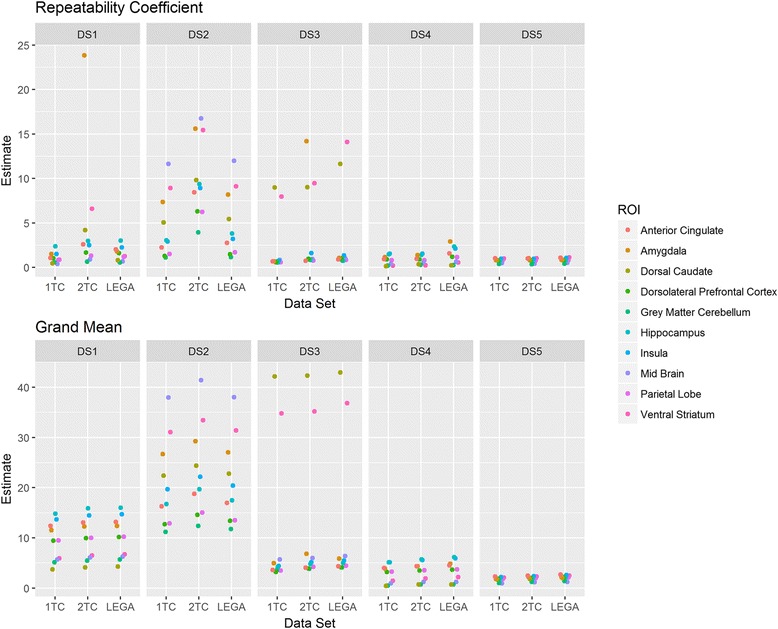
Repeatability coefficient (RC) and grand mean across the considered datasets (upper panel and lower panel respectively). Each point in the plot, for a given dataset, represents a particular region of interest (ROI) so that the plot represents the variability of RC across the considered ROIs. For each data set, DS1, DS2, DS3, DS4, and DS5, there were three quantification methods investigated (1, 2, and L denote one-tissue compartment model, two-tissue compartment model, and likelihood estimation in graphical analysis, respectively)

Figure 3 shows RC as an unscaled index of agreement along with the grand mean μ derived from random effects ANOVA (Eq. 1). The outlying ROIs appear as influential points in the plots.

A key utility of the test-retest metrics is selecting a tracer among many for a particular target. For example, [^11^C]WAY-100635 and [^11^C]CUMI-101 are both tracers for the serotonin 1A receptor. The ICC, PTRT, and WSCV show lower test-retest variability for [^11^C]CUMI-101 compared to [^11^C]WAY-100635 (Figs. 1 and 2), indicating that [^11^C]CUMI-101 considering only the test-retest repeatability aspect would be preferred of the tracer, for the serotonin 1A receptor.

**Fig. 4:**
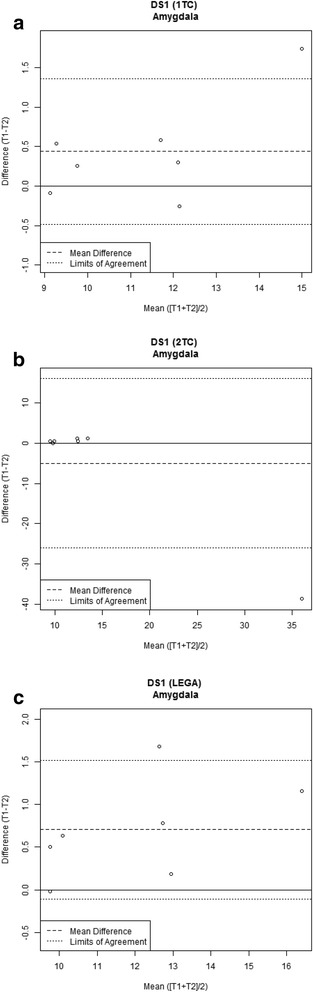
Bland-Altman plots of the [^11^C]CUMI-101 dataseDS1 for **a** 1TC model, **b** 2TC model, and **c** LEGA approach

In order to investigate various real-life scenarios, a graphical representation of the data by means of the Bland-Altman plots is shown for a particular dataset ([^11^C]CUMI-101) and a particular ROI (amygdala) across different quantification strategies (Fig. 4). Ninety-five percent of differences between test-retest measures are expected to lie between the limits of agreement, and these lines indicate if the two measures can be interchanged without altering the clinical interpretation [15].

The other metrics obtained from this particular dataset and ROI are reported in Table 2. From Fig. 4a, c as well as Table 2, it can be seen that the test-retest repeatability for the 1TC model and LEGA is very good across both scaled indices of agreement (WSCV and PTRT are about 5%, and ICC is higher than 0.93) and where the Bland-Altman plots show random variation across the sampling range, albeit with small bias for both methods. Good repeatability can also be observed in the ratio of the RC and grand mean μ, as this ratio is obtained as WSCV scaled by a constant factor. For the 2TC model, however, the test-retest repeatability is quite poor. As shown in the Bland-Altman plot in Fig. 4b, there is an influential, outlying observation for a particular subject. This may be due to poor identifiability of one of the four kinetic rate parameters of the 2TC model, which results in unreasonably high value for that ROI V_T_ and thus may cause deterioration of the overall test-retest metrics. Notably, the PTRT appears to be less sensitive to the outlier. This may be explained by the local as opposed to global scaling of the PTRT and WSCV, respectively. This potential insensitivity of PTRT to outliers values underscores the utility of Bland-Altman plots to visualize test-retest data. This result also strongly underscores the value of reporting more than just PTRT in PET test-retest studies, since this metric attenuates a poor test-rest datapoint, while ICC and WSCV appropriately highlight its influence.

**Table 2:** Agreement indices for amygdala in the [^11^C]CUMI-101 dataset for the three considered quantification approaches

Tracer	Quantification strategy	WSCV	PTRT	ICC	RC	Grand mean
[^11^C]CUMI-101	LEGA	0.05	0.06	0.93	1.79	12.40
[^11^C]CUMI-101	1TC	0.05	0.04	0.94	1.49	11.52
[^11^C]CUMI-101	2TC	0.70	0.20	0.25	23.87	12.28

## Discussion

Our main goal was to investigate current approaches to the evaluation of test-retest experiments in PET brain imaging from a statistical point of view and to provide insights and guidance for using indices of agreement in addition to the typically reported PTRT metric. In this evaluation, the random effects ANOVA model underpins the rationale for most metrics and we found it to be a useful model for brain PET imaging, as it describes and quantifies the test-retest PET experiments in a succinct way, while at the same time capturing various random variations present in the data. With respect to random effects ANOVA, three metrics obtained from the model (ICC, RC, and WSCV) reveal several aspects of the data. The ICC provides information about distinguishability of the subjects [23]. As ICC is a ratio of between-subject variance to total variance, it quantifies the agreement of the test-retest readings (given by the within-subject standard deviation (WSSD)) relative to the spread of the subjects (characterized by between-subject standard deviation). The higher the between-subject variability is, the better the distinguishability. As ICC depends on the between-subject variability expressed by the between-subject deviation, it has been pointed out that care needs to be paid to comparisons of the ICC across groups for which the between-subject variability may be different [23]. WSCV provides information about the agreement between test-retest readings with respect to the overall signal (estimated as population mean from the random effects ANOVA model). RC is an unscaled index of agreement, reflecting agreement between the test-retest readings proportional to the WSSD (which is estimated as a square root of the within-subject mean sum of squares or WSMSS).

In PET imaging literature, several test-retest outcome metrics are commonly reported, but there has been no general consensus as to which outcome metrics should be used. We found it useful to classify the metrics based on the underlying statistical model, such as random effects ANOVA vs. other metrics. The most popular metrics based on random effect ANOVA are ICC and WSMSS [8–12, 24, 25]*.* WSMSS is directly related to the RC, as square root of WSMSS and is an estimate of the WSSD. WSCV, which also ensues for random effect ANOVA model, is only rarely reported in test-retest studies in PET brain imaging [11]*.* In PET test/retest studies, ICC is usually calculated assuming a one-way ANOVA (4). However, in some cases, a two-way mixed effect model has also been applied [26]*.* Since typical test/retest studies consist of two images per subject, we generally recommend calculating ICC according to the one-way model.

The most commonly used test-retest metric in PET imaging is PTRT (reported virtually in all PET imaging studies with test-retest experiment). PTRT is obtained from mean normalized differences of test-retest samples. With respect to the random effects ANOVA model, PTRT does not estimate any parameter or function of parameters of the model. Using a first order Taylor expansion (see also Appendix), it can be shown that the mean normalized differences are akin to taking log transform of the data. Therefore, it is expected that the PTRT will not be as sensitive to outliers, as these will be scaled “locally” by the corresponding test-retest mean. Also, due to local scaling, the spread of PTRT is small compared to ICC where the scaling is global. This may significantly underestimate the test-retest repeatability measured with PTRT as seen in the analysis of [11C]CUMI dataset (Table 2). Both PTRT and WSCV provide an intuition to the tracer’s limit on detecting differences (e.g., a difference smaller than PTRT and WSCV is unlikely to be detected). The overall rank ordering of regions in terms of test-retest reliability is similar between PTRT and WSCV. Due to inherent small sample size in PET reliability experiments, confidence intervals for the test-retest metrics will generally be fairly wide. Thus, small differences in these measures may not be meaningful. As a general recommendation, the random effect ANOVA model is a useful model for the PET test-retest studies and therefore measures ensuing from it should be reported together with the PTRT, in the case of two repeated measures (one test and one retest). Although more than two repetitions for the PET imaging are not typical, it is worth to note that PTRT is not straightforwardly generalizable for more than two test/retest periods, whereas the ANOVA indices can be applied naturally regardless of the number of repeated observations.

Test-retest metrics that are directly derived from the random ANOVA model (WSCV, ICC, and RC) can be also used for sample size calculation when planning a study that involves multiple PET scans per subject. A method for sample size calculation for ICC was suggested in [27], which is based on determination of necessary sample size to achieve pre-specified precision of ICC given by a corresponding confidence interval width. This approach can be used in a straightforward way also for the WSCV and RC indices, but not for the PTRT. We emphasize that while these summaries are quite valuable for planning studies that involve multiple PET scans per subject, they are not directly relevant for planning cross-sectional studies. For example, for a pre-post study design, within-subject standard deviation obtained from a test-retest experiment may be used for sample size calculation given an assumed effect size (mean difference between pre- and post- periods) as shown in [28].

Bland-Altman plots represent a mainstay in the graphical display of test-retest data. However, they are rarely used in PET brain imaging [29]. Bland-Altman plots should be used as a first step in the analysis as they may be helpful in better understanding the dependence of variability on the signal strength as well as potential bias between test and retest measurements.

When characterizing test-retest properties of a particular tracer, one may aim at an overall measure across several ROIs or at a region-specific measure of reliability in a priori regions with hypothesized or confirmed biological relevance to the population and/or application at hand. In our investigation, we found that some ROIs may exhibit better performance than others, so ROI-wise comparisons are worth considering. In addition, various ROIs may show different uptake characteristics that influence their noise properties (e.g., high-binding vs. low-binding ROIs), and in that case, test-retest properties could be investigated region-by-region; however, pooling all ROIs into an aggregate test-retest metric may also be carried out if there is an application specific requirement. The difference in ROI-size influences the noise in the region which is the cause of test-retest repeatability metric. Thus, the ROI size will not have an impact on the conclusions drawn from test-retest repeatability metrics if the image processing is performed in a uniform fashion across studies, which was the case in the datasets chosen for this paper.

All the scaled metrics will be useful to compare repeatability of the same ROIs from different tracers as well as different ROIs of the same tracer. As seen in case of [^11^C]CUMI-101 and [^11^C]WAY-100635 for the serotonin 1A receptor; all things being equal, these repeatability metrics can help choose the tracer for a given target.

## Conclusions

Random effects ANOVA is a useful model for PET brain imaging test-retest studies. The metrics that ensue from this model such as ICC, RC and WSVC are recommended to be reported along with the percent test-retest metric as they capture various sources of variability in the PET test-retest experiments in a succinct way.

## Appendix

Estimation of the parameters in test-retest experiment (estimators denoted by hat):

$$ {\displaystyle \begin{array}{l}{\widehat{\sigma}}_e^2=\mathrm{WSMSS}=\frac{1}{n}\sum \limits_{i=1}^n\sum \limits_{j=1}^2{\left({y}_{ij}-{\overline{y}}_i\right)}^2\\ {}\mathrm{BSMSS}=\frac{1}{n-1}\sum \limits_{i=1}^n{\left({\overline{y}}_i-\widehat{\mu}\right)}^2\\ {}\widehat{\mu}=\frac{1}{n}\sum \limits_{i=1}^n{\overline{y}}_i\\ {}{\widehat{\sigma}}_S^2=\frac{\left(n-1\right)\mathrm{BSMSS}-n\kern0.5em \mathrm{WSMSS}}{\left(n-1\right)\mathrm{BSMSS}+n\kern0.5em \mathrm{WSMSS}}\\ {}\\ {}\mathrm{R}\widehat{\mathrm{C}}=\sqrt{2}\kern0.5em {z}_{1-\alpha /2}{\widehat{\sigma}}_e\end{array}} $$

Where WSMSS and BSMSS are within and between mean sum of squares, respectively.

Also, confidence intervals for the estimates of ICC and WSCV are available (see [14] and [30], respectively).

For the RC under the one-way random ANOVA model, the confidence limits of the exact 100(1−α)% CI can be obtained as follows (*r* is number of repetitions, typically *r* = 2):

$$ {\displaystyle \begin{array}{l}\mathrm{R}\widehat{\mathrm{C}}{\mathrm{C}}_L={z}_{1-\alpha /2}\sqrt{2n\left(r-1\right)\mathrm{WSMSS}/{\chi}_{n\left(r-1\right)}^2\left(1-\alpha /2\right)}\\ {}\mathrm{R}\widehat{\mathrm{C}}{\mathrm{C}}_U={z}_{1-\alpha /2}\sqrt{2n\left(r-1\right)\mathrm{WSMSS}/{\chi}_{n\left(r-1\right)}^2\left(\alpha /2\right)}\\ {}{\mathrm{C}\mathrm{I}}_{\mathrm{WIDTH}}=\mathrm{R}\widehat{\mathrm{C}}{\mathrm{C}}_U-\mathrm{R}\widehat{\mathrm{C}}{\mathrm{C}}_L\end{array}} $$

where ${\chi}_d^2\left(\alpha \right)$ is an α quantile of *χ*^2^ distribution with *d* degrees of freedom.

Mean normalized difference as a Taylor expansion-based approximation of log transformed differences.

Consider two real numbers *y*2 and *y*1 (e.g. they could represent two test-retest measurements):

Then, their difference (diff), mean normalized difference (mdiff), and log difference (ldiff) are defined as follows:

$$ {\displaystyle \begin{array}{l}\mathrm{diff}={y}_2-{y}_1\\ {}\mathrm{mdiff}=\left({y}_2-{y}_1\right)\frac{1}{\left({y}_2+{y}_1\right)/2}=\frac{2\mathrm{diff}}{\left({y}_2+{y}_1\right)}\\ {}\mathrm{ldif}f=\log {y}_2-\log {y}_1=\log \frac{y_2}{y_1}\end{array}} $$

Let *R* be defined as follows:

$$ R=1-\frac{y_2}{y{}_1} $$

Then expressing mdiff and ldiff in terms of R and expanding them as the Taylor series in terms of *R* we obtain the following:

$$ {\displaystyle \begin{array}{l}\mathrm{mdiff}=R-\frac{R^2}{2}+\frac{R^3}{4}-\frac{R^4}{8}+\dots {\left(-1\right)}^{i-1}\frac{R^i}{2^{i-1}}+\dots \\ {}\mathrm{ldiff}=R-\frac{R^2}{2}+\frac{\begin{array}{l}\\ {}{R}^3\end{array}}{3}-\frac{R^4}{4}+\dots {\left(-1\right)}^{i-1}\frac{R^i}{i}+\dots \\ {}\end{array}} $$

We observe that the first two terms of the Taylor expansion for mdiff and ldiff are identical, and they differ at the higher order terms greater than 2. Therefore, mdiff can be considered an approximation of ldiff.
